# ER Stress Induces Anabolic Resistance in Muscle Cells through PKB-Induced Blockade of mTORC1

**DOI:** 10.1371/journal.pone.0020993

**Published:** 2011-06-16

**Authors:** Louise Deldicque, Luc Bertrand, Amy Patton, Marc Francaux, Keith Baar

**Affiliations:** 1 Université catholique de Louvain, Institute of Neuroscience, Research Group in Muscle and Exercise Physiology, Louvain-la-Neuve, Belgium; 2 Research Centre for Exercise and Health, Department of Biomedical Kinesiology, K.U. Leuven, Leuven, Belgium; 3 Université catholique de Louvain, Institut de Recherche Expérimentale et Clinique, Pole of Cardiovascular Research, Woluwe-Saint-Lambert, Belgium; 4 Department of Neurobiology, Physiology and Behaviour, University of California Davis, Davis, California, United States of America; McMaster University, Canada

## Abstract

**Background:**

Anabolic resistance is the inability to increase protein synthesis in response to an increase in amino acids following a meal. One potential mediator of anabolic resistance is endoplasmic reticulum (ER) stress. The purpose of the present study was to test whether ER stress impairs the response to growth factors and leucine in muscle cells.

**Methods:**

Muscle cells were incubated overnight with tunicamycin or thapsigargin to induce ER stress and the activation of the unfolded protein response, mTORC1 activity at baseline and following insulin and amino acids, as well as amino acid transport were determined.

**Results:**

ER stress decreased basal phosphorylation of PKB and S6K1 in a dose-dependent manner. In spite of the decrease in basal PKB phosphorylation, insulin (10–50 nM) could still activate both PKB and S6K1. The leucine (2.5–5 mM)-induced phosphorylation of S6K1 on the other hand was repressed by low concentrations of both tunicamycin and thapsigargin. To determine the mechanism underlying this anabolic resistance, several inhibitors of mTORC1 activation were measured. Tunicamycin and thapsigargin did not change the phosphorylation or content of either AMPK or JNK, both increased TRB3 mRNA expression and thapsigargin increased REDD1 mRNA. Tunicamycin and thapsigargin both decreased the basal phosphorylation state of PRAS40. Neither tunicamycin nor thapsigargin prevented phosphorylation of PRAS40 by insulin. However, since PKB is not activated by amino acids, PRAS40 phosphorylation remained low following the addition of leucine. Blocking PKB using a specific inhibitor had the same effect on both PRAS40 and leucine-induced phosphorylation of S6K1.

**Conclusion:**

ER stress induces anabolic resistance in muscle cells through a PKB/PRAS40-induced blockade of mTORC1.

## Introduction

Amino acids activate mTORC1 (mammalian target of rapamycin complex 1) by promoting the exchange of GDP for GTP in the RagA/B GTPase proteins [1], [2]. When RagA/B is bound to GTP, mTORC1 is recruited to the lysosome through its association with the Ragulator scaffolding complex [3]. At the lysosome, mTORC1 interacts with Rheb (ras homologous enriched in brain) and is activated [3]. The requirement of PI-3 (phosphatidyl-inositol-3) kinase and PKB (protein kinase B/akt) for the activation of mTORC1 by amino acids is controversial [4]–[7] and it has been suggested that this process could be independent of PKB [8]. However, in some pathological states and during aging, the response to amino acids can be altered. The inability to increase protein synthesis in response to an increase in amino acids following a meal, irrespective of the availability of insulin, insulin-like growth factor 1, and growth hormone has been called anabolic resistance [9]. In skeletal muscle, this anabolic resistance is thought to contribute to the loss of muscle mass in aging [10]; immobilization [11]; and high-fat feeding/obesity [12]. In spite of the fact that PKB is not necessarily required for the amino acid-induced increase in protein synthesis and mTORC1 activation, anabolic resistance is characterized primarily by decreased phosphorylation of PKB.

mTORC1 is the central molecular player in anabolic resistance. mTORC1 is composed of mTOR (a Ser/Thr protein kinase), raptor (regulatory associated protein of mTOR) and mLST8/GβL (G protein beta subunit-like protein) and is sensitive to the macrolide rapamycin [13], [14]. It regulates cell growth and protein synthesis through the phosphorylation of 4EBP1 (initiation factor 4E binding protein) and S6K1 (ribosomal protein S6 kinase). Activation of S6K1 and 4E-BP1 requires sequential phosphorylation events. For S6K1, phosphorylation of Ser/Thr residues in the autoinhibitory domain, such as at Thr421 and Ser424, is required for altering its conformation and making Thr389 and Thr229 available for phosphorylation, thereby fully activating S6K1 [15]. 4E-BP1 also possesses many different phosphorylation sites [16]. Thr37 and Thr46 phosphorylation serves as a priming step for subsequent phosphorylation at Ser65 and Ser70 in the carboxy-terminus that results in release from eIF4E (eukaryotic initiation factor 4E). mTORC1 is the primary kinase for the Thr389 site in S6K1 and the Thr37/46 sites in 4E-BP1. The other mTOR complex, called mTORC2, is composed of mTOR, rictor (rapamycin-insensitive companion of mTOR), mSIN1 (mammalian stress-activated protein kinase-interacting protein), mLST8/GβL and PRR5 (proline rich protein 5), and is resistant to inhibition by rapamycin [17], [18]. Activation of mTORC2 has been shown to regulate PKB Ser473 phosphorylation [19]. PKB, in turn, can regulate the activity of mTORC1 in three ways: 1) PKB can directly phosphorylate mTORC1 [20]; 2) PKB can phosphorylate and inhibit TSC2 (tuberous sclerosis complex 2) a GTPase activating protein that targets Rheb [21]; and 3) PKB can phosphorylate PRAS40 (prolinerich Akt substrate 40), an allosteric inhibitor of mTORC1 [22], [23]. When unphosphorylated, PRAS40 binds to raptor and prevents the association of mTORC1 with its downstream targets. Phosphorylation of PRAS40 by PKB on Thr246 alters its conformation such that a TOS (TOR signaling motif) motif is unmasked. mTORC1 then phosphorylates PRAS40 on Ser183 and Ser221, resulting in dissociation of PRAS40 and allosteric activation of mTORC1 [22].

One potential mediator of anabolic resistance is endoplasmic reticulum (ER) stress [24]–[28]. Periods of high lipids, glucose deprivation, or increased synthesis of secretory proteins lead to the accumulation of unfolded or misfolded proteins within the ER lumen [29]. To cope with this ER stress, cells activate the unfolded protein response, a series of events that serve to restore ER function [30]. The unfolded protein response has three main effectors: ATF6 (activating transcription factor 6); IRE1α (inositol-requiring enzyme 1 alpha); and PERK (protein kinase R-like ER protein kinase). In the basal (inactive) state, each of these factors associates with the protein chaperone BiP/GRP78 (binding protein/glucose regulated-protein 78), a member of the Hsp70 (heat shock protein 70) family [31]. Upon accumulation of unfolded/misfolded proteins, ATF6, IRE1α, and PERK are released from BiP/GRP78 and become activated [30]. The best characterized downstream effect of ATF6, IRE1α, and PERK release is the induction of genes, such as XBP1 (X box binding protein 1), CHOP (CCAAT/enhancer binding protein (C/EBP) homologous protein) and ATF4 (activating transcription factor 4), which increase the protein-folding capacity of the cell [30]. Concomitant with the increase in protein folding, there is a decrease in protein synthesis that is in part due to the phosphorylation and inhibition of eIF2α [32], and might also be dependent on mTORC1 [28]. When the unfolded protein response fails, the cells undergo apoptosis indicating that the unfolded protein response is essential for normal cellular function [33].

In the present study, we sought to determine whether ER stress could induce anabolic resistance in muscle cells. Consistent with this hypothesis, low levels of ER stress were sufficient to prevent the activation of mTORC1 by leucine, assessed by a decrease in S6K1 phosphorylation on Thr389, whereas at the same level of ER stress insulin could still activate mTORC1 normally. The inability to activate mTORC1 was not due to a lack of leucine transport, but rather to the ER stress-induced decrease in basal PKB phosphorylation resulting in PRAS40 hypophosphorylation and allosteric inhibition of mTORC1.

## Materials and Methods

### Cell cultures

C2C12 murine skeletal muscle myoblasts (ATCC, USA) were seeded in 150 mm-diameter culture dishes and grown in Dulbeccos's Modified Eagle Medium (DMEM, Life technologies) supplemented with 10% fetal bovine serum and 1% penicillin/streptomycin (5000 U/5000 µg/ml). Cells were then plated in 6-well plates until 90% confluent. At this time, the proliferation medium was replaced by a differentiation medium containing 2% horse serum and 1% penicillin/streptomycin (5000 U/5000 µg/ml). After 96 h of differentiation, tunicamycin (MP Biomedicals) or thapsigargin (Tocris Bioscience) was added for 17 h in serum-free DMEM before stimulation of the mTORC1 pathway with insulin (10–50 nM) for 15 min or with leucine (2.5–5 mM) for 30 min. Tunicamycin and thapsigargin were used as they are well-documented chemical inducers of ER stress by blocking N-glycosylation and calcium entry into the ER, respectively. For PKB inhibition experiments, cells were serum-starved for 16 h to maintain the cells in the same conditions as those described for tunicamycin and thapsigargin experiments. PKB inhibitor (100 nM–1 µM), also known as Akt Inhibitor XIII or Akti2 (Calbiochem), was then added 1 h before leucine (2.5–5 mM) stimulation for 30 min. At the end of the incubation period, cells were harvested and cell lysates were immediately frozen at −80°C for subsequent analyses or cells were immediately used for leucine uptake measurements. All experiments were performed at least in duplicate.

### Protein extraction, SDS/PAGE and immunoblotting

Cells were rinsed once with PBS and harvested in a lysis buffer containing 20 mM Tris, pH 7.0, 270 mM sucrose, 5 mM EGTA, 1 mM EDTA, 1% Triton X-100, 1 mM sodium orthovanadate, 50 mM sodium β-glycerophosphate, 5 mM sodium pyrophosphate, 50 mM sodium fluoride, 1 mM DTT (1,4-dithiothreitol) and a protease inhibitor cocktail containing 1 mM EDTA (Roche Applied Science). The homogenates were then centrifuged for 10 min at 10,000 g and the supernatants were immediately stored at −80°C. Protein concentration was determined using the DC protein assay kit (Bio-Rad Laboratories).

Cell lysates (15 µg) were combined with Laemmli sample buffer and separated by SDS/PAGE. After electrophoretic separation at 40 mA for 1 h, the proteins were transferred to nitrocellulose membrane at 100 V for 1 h for western blot analysis. Membranes were then incubated in a 5% Blotto solution. Subsequently, membranes were incubated with the following antibodies overnight at 4°C: eEF2; S6K1; phospho-S6K1 Thr389 and Thr421/Ser424; phospho-PKB Ser473 and Thr308; phospho-PRAS40 Thr246; phospho-mTOR Ser2448; phospho-PDK1 Ser241; phospho-4E-BP1 Thr37/46; phospho-GSK3 Ser9/21; BiP; IRE1α; phospho-PERK Thr980; and phospho-eIF2α Ser51. All antibodies were from Cell Signaling except phospho-S6K1 Thr421/Ser424 and S6K1 that were acquired from Santa Cruz and phospho-PRAS40 acquired from Invitrogen.

Membranes were washed in TBST and incubated for 1 h at room temperature in a secondary antibody conjugated to horseradish peroxidase. After an additional 3 washes, chemiluminescence detection was carried out using an Enhanced Chemiluminescent Western blotting kit (ECL Plus, GE Healthcare). The films were then scanned on an ImageScanner using the Labscan software and quantified with the Image Master 1D Image Analysis Software (GE Healthcare). Equal loading was checked by probing membranes with an anti-eEF2 as previous experiments showed that the different treatments used in the present study did not affect eEF2 expression.

### RNA extraction and quantitative Real-Time PCR

Total RNA from the cells of one 35 mm-well (6-well plates) was extracted with 0.5 ml TriPure reagent according to the instructions provided by the manufacturer (Roche Diagnostics). RNA was quantified by spectrophotometry (260 nm) and its concentration adjusted to 1 µg/µl using RNase-free water. cDNA was prepared by reverse transcription of 1 µg total RNA using the reverse transcription system (Promega). SYBR®Green (Sigma Aldrich) was used for real-time PCR detection using an Eppendorf Light Cycler PCR machine. Real-time PCR primers were designed (Table 1) for mouse CHOP, ATF4, spliced (s) XBP1, unspliced (u) XBP1, TRB3, LAT1, SNAT2, 4F2hc, ASC1, cMyc, MAD1 and GAPDH. Specific primers were designed to recognize the spliced, or active, form of XBP1 (XBP1s) versus the unspliced form (XBP1u). GAPDH was used as the reference gene and the Ct for GAPDH was unchanged by the treatments. All samples were run in triplicate in a single 96-well reaction plate and the data were analyzed according to the 2^−ΔΔCt^ method. The identity and purity of the amplified product was checked through analysis of the melting curve carried out at the end of amplification.

**Table 1:** Sequences of primers used for mRNA quantification by real-time RT-PCR.

	Forward	Reverse
CHOP	CCT AGC TTG GCT GAC AGA GG	CTG CTC CTT CTC CTT CAT GC
ATF4	GAG CTT CCT GAA CAG CGA AGT G	TGG CCA CCT CCA GAT AGT CAT C
XBP1s	GAG TCC GCA GCA GGT G	GTG TCA GAG TCC ATG GGA
XBP1u	AAG AAC ACG CTT GGG AAT GG	ACT CCC CTT GGC CTC CAC
TRB3	TGT GAG AGG ACG AAG CTG GTG	TCG TGG AAT GGG TAT CTG CC
LAT1	GCT GCC TGC ATC TGT CTC TTA AC	CTG CAA TCA GCG CCA ACA C
SNAT2	TCT TGT CCT TCA CCA ATT TGC TC	CCC CAA TGA ACC CGA AGA TG
4F2hc	CTT TCA CAT CCC AAG ACC TGT AAG	GAG GAA GAC AGT GCA TGG AAG TC
ASC1	CCA CGC GCA TCC AGG TTA	GGT GTC ATC CAG AAG GCG AAG
cMyc	CAC CAG CAG CGA CTC TGA AGA	ATG AGC CCG ACT CCG ACC
MAD1	CAA GCC GAC ACA CCA CTC TGA	ACG CTG TCC ATC CGA GTC C
GAPDH	TGG AAA GCT GTG GCG TGA T	TGC TTC ACC ACC TTC TTG AT

CHOP, C/EBP (CCAAT/enhancer binding protein) homologous protein; ATF4, activating transcription factor 4; XBP1s, spliced X Box binding protein 1; XBP1u, unspliced X Box binding protein 1; TRB3, tribbles homolog 3; LAT1, L-type amino acid transporter 1; SNAT2, sodium-coupled neutral amino acid transporter 2; 4F2hc, 4F2 heavy chain; ASC1, sodium-independent alanine-serine-cysteine transporter 1; MAD1, mitotic arrest-deficient 1; GAPDH, glyceraldehyde-3-phosphate dehydrogenase.

### Leucine uptake measurement

At the end of the 17 h incubation with tunicamycin and thapsigargin, cells were washed with Hepes-buffered saline (HBS: 140 mM NaCl, 20 mM Hepes, 5 mM KCl, 2.5 mM MgSO_4_ and 1 mM CaCl_2_, pH 7.4), pre-warmed at 37°C. Cells were incubated for 1 min with 0.25 µCi (or 0.5 µCi) [^14^C]leucine diluted in 500 µl HBS containing 10 µM (or 1 mM) leucine. The doses of cold leucine were chosen to be below the Km of the transporter and about ∼10 times higher (Km = ∼100 µM, [34], [35]). Medium was aspirated before washing cells 2 times with 0.9% (w/v) ice-cold NaCl containing 100 mM leucine. Cells were subsequently lysed in 50 mM NaOH, and radioactivity was quantified using a Beckman LS 6000IC scintillation counter. Specific activity was determined in each well by measuring radioactivity in the incubation medium. Protein concentration in cell lysates was determined using the DC Protein Assay (Bio-Rad). Leucine uptake was reported to protein content and expressed as fold control. The experiment was repeated three times and the results are presented as the mean.

## Results

### ER stress is induced proportionally to tunicamycin and thapsigargin concentrations

**Figure 1:**
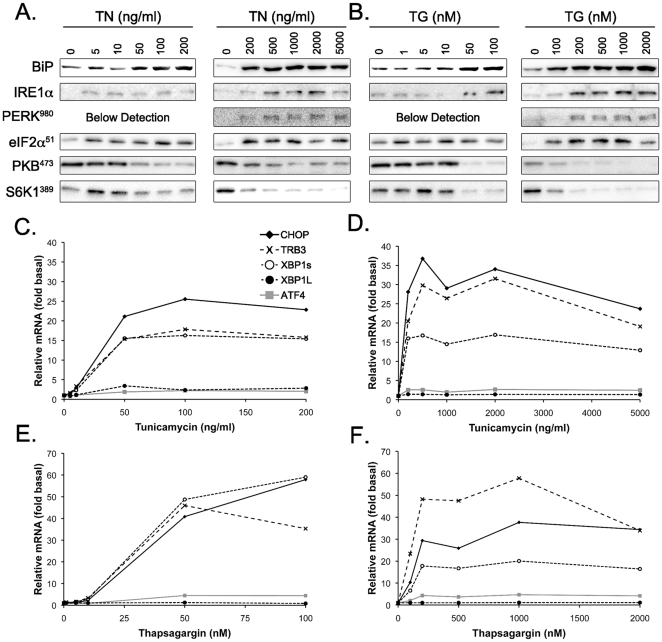
Dose-response curves of tunicamycin and thapsigargin on ER stress markers and mTORC1 pathway. Effect of increasing doses of tunicamycin (A) and thapsigargin (B) on BiP and IRE1α expression and PERK (Thr980), eIF2α (Ser51), PKB (Ser473) and S6K1 (Thr389) phosphorylation after 17 h incubation. Effect of low doses of tunicamycin (C) and thapsigargin (E) and high doses of tunicamycin (D) and thapsigargin (F) on CHOP, ATF4, XBP1s, XBP1l and TRB3 mRNA level after 17 h incubation. TN, tunicamycin; TG, thapsigargin; BiP, binding protein; IRE1α, inositol-requiring enzyme 1 alpha; PERK, protein kinase R-like ER protein kinase; eIF2α, eukaryotic initiation factor 2 alpha; PKB, protein kinase B; S6K1, ribosomal protein S6 kinase 1; CHOP, C/EBP (CCAAT/enhancer binding protein) homologous protein; ATF4, activating transcription factor 4; XBP1s, spliced X Box binding protein 1; XBP1u, unspliced X Box binding protein 1; TRB3, tribbles homolog 3.

Increasing doses of tunicamcyin (0–5000 ng/ml, Figure 1A) and thapsigargin (0–2000 nM, Figure 1B) increased BiP and IRE1α protein and the phosphorylation of PERK (Thr980) and eIF2α (Ser51) whereas the phosphorylation of PKB (Ser473) and S6K1 (Thr389) decreased. The decrease in phospho-PKB and phospho-S6K1 correlated with the increase in BiP suggesting a common mechanism (Pearson product moment correlation; r = −0.85, P<0.05). Tunicamycin (Figure 1C and D) and thapsigargin (Figure 1E and F) increased CHOP, XBP1s and TRB3 mRNA more than 30-fold. The activation of TRB3 was of particular interest since it is known to impair PKB activation [36]. ATF4 mRNA was more than doubled by tunicamycin and thapsigargin whereas XBP1l only increased with tunicamycin (Figure 1C–F).

### ER stress decreases leucine-induced phosphorylation of PKB and S6K1

**Figure 2:**
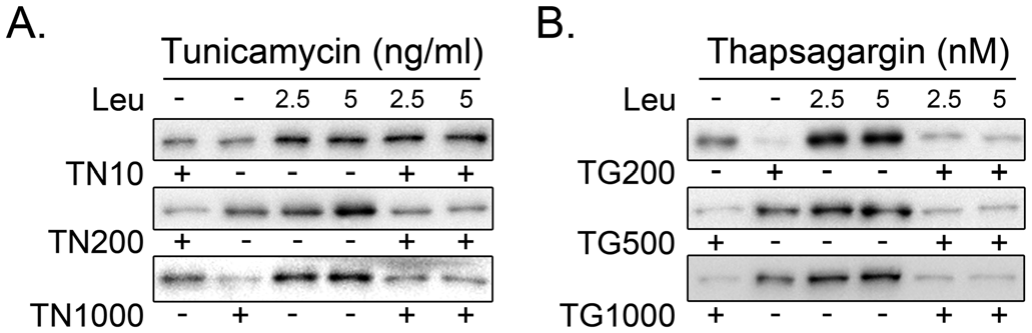
Effect of tunicamycin and thapsigargin on leucine-induced phosphorylation of S6K1 (Thr389). Cells were incubated for 17 h with different doses of tunicamycin (A) or thapsigargin (B) before stimulation with 2.5 mM or 5 mM leucine for 30 min. TN, tunicamycin; TG, thapsigargin; S6K1, ribosomal protein S6 kinase 1; Leu, leucine.

Tunicamycin (Figure 2A) and thapsigargin (Figure 2B) not only decreased basal phosphorylation of PKB (Ser473) and S6K1 (Thr389) but also repressed leucine-induced phosphorylation of S6K1 (Thr389) at all concentrations from 100 ng/ml for tunicamycin and 200 nM for thapsigargin.

### ER stress decreases insulin-induced phosphorylation of PKB and S6K1

**Figure 3:**
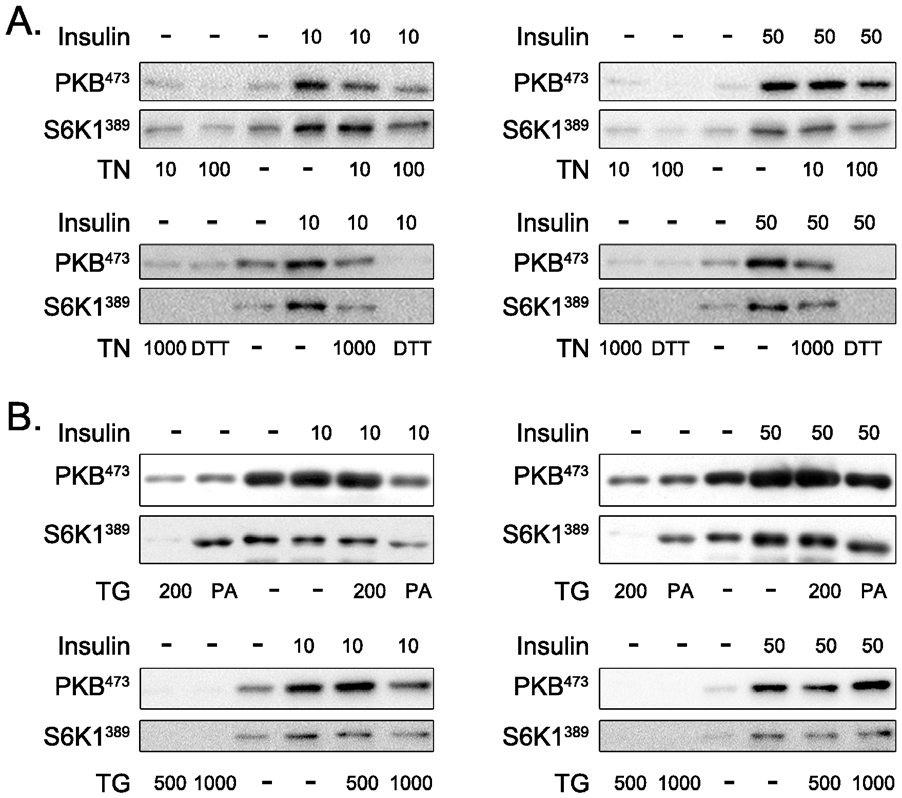
Effect of tunicamycin and thapsigargin on insulin-induced phosphorylation of PKB (Ser473) and S6K1 (Thr389). Cells were incubated for 17 h with different doses of tunicamycin (A) or thapsigargin (B) before stimulation with 10 nM or 50 nM insulin for 15 min. TN, tunicamycin; TG, thapsigargin; PKB, protein kinase B; S6K1, ribosomal protein S6 kinase 1; Ins, insulin; DTT, dithiotreitol; PA, palmitic acid.

Tunicamycin (Figure 3A) and thapsigargin (Figure 3B) also repressed insulin-induced phosphorylation of PKB and S6K1, but this repression was not complete. The effects of tunicamycin were stronger than thapsigargin at all concentrations. Insulin-induced phosphorylation of PKB was already partially repressed by 10 ng/ml tunicamycin, considered as a low dose according to previous reports [37], whereas 1000 nM thapsigargin, a large dose [38], was necessary to observe a similar inhibition. Dithiothreitol (1 µM, Figure 3A) and palmitic acid (1 mM, Figure 3B) were used as additional controls to which tunicamcyin and thapsigargin could be compared, as they are also known to induce ER stress. It is important to note that the concentration of TG required to reduce PKB/S6K1 phosphorylation following leucine stimulation was much less than after insulin stimulation (200 nM vs. 1000 nM).

### Leucine transport is not involved in the impairment of mTORC1 activity by ER stress

**Figure 4:**
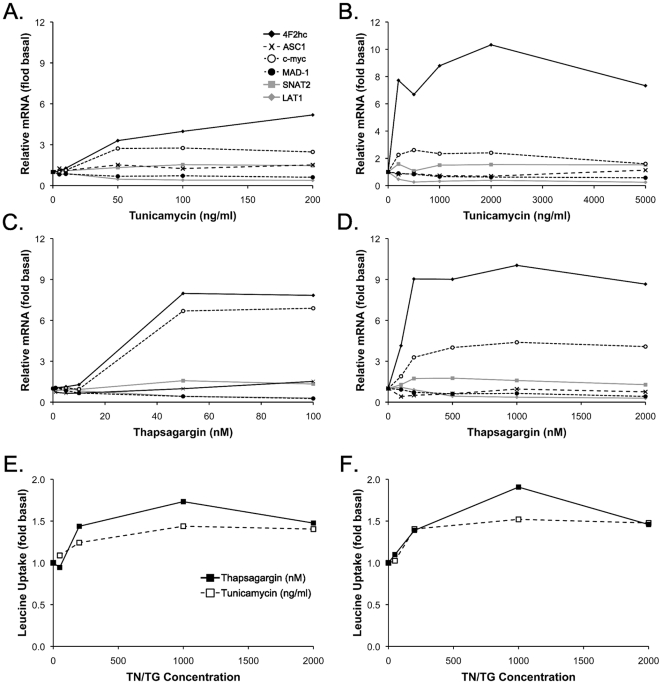
Dose-response curves of tunicamycin and thapsigargin on amino acid transport regulation and leucine uptake. Effect of low doses of tunicamycin (A) and thapsigargin (C) and high doses of tunicamycin (B) and thapsigargin (D) on the mRNA level of amino acid transporters and amino acid transporters regulators after 17 h incubation. Effect of increasing doses of tunicamycin and thapsigargin on the uptake of 10 µM (E) and 1 mM (F) leucine. TN, tunicamycin; TG, thapsigargin; LAT1, L-type amino acid transporter 1; SNAT2, sodium-coupled neutral amino acid transporter 2; 4F2hc, 4F2 heavy chain; ASC1, sodium-independent alanine-serine-cysteine transporter 1; MAD1, mitotic arrest-deficient 1; leu, leucine.

We first hypothesized that the greater sensitivity of leucine stimulation to ER stress was due to a decrease in leucine uptake. To test this hypothesis, we analysed the expression of transporters known to be directly or indirectly involved in leucine transport in skeletal muscle: LAT1, SNAT2 and ASC1, as well as adaptors and regulators of these transporters: 4F2hc, cMyc, and MAD. Tunicamycin and thapsigargin increased expression of 4F2hc and cMyc and decreased the expression of LAT1 and MAD1 in a dose-dependent manner (Figure 4A–D). Since LAT1, the primary leucine transporter in muscle, expression was decreased, leucine uptake was determined at two different concentrations based on the transport kinetics (Km = ∼100 µM, [34], [35]): a non-saturating dose (10 µM, Figure 4E) and a saturating dose (1 mM, Figure 4F). Contrary to our hypothesis, at both concentrations, leucine uptake was slightly increased by tunicamycin (∼40%) and thapsigargin (∼60%).

### PRAS40 phosphorylation by PKB is required for amino acid-induced mTORC1 activity

**Figure 5:**
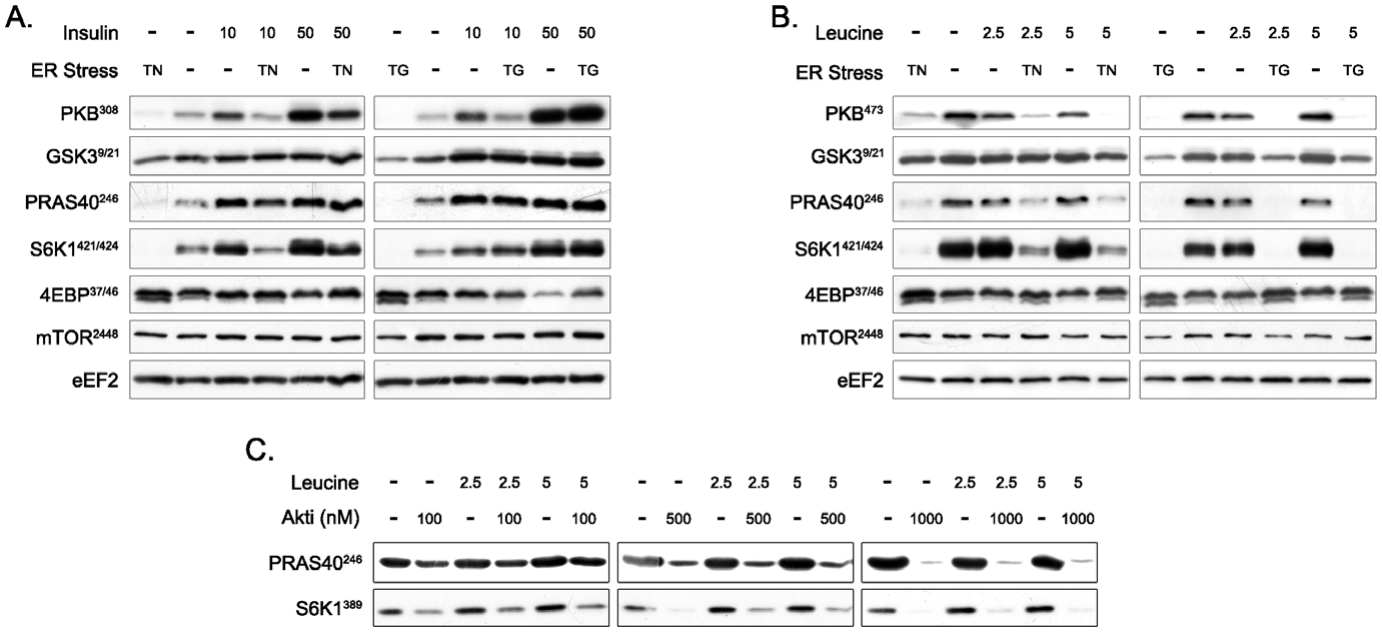
Effect of tunicamycin and thapsigargin on insulin or leucine-induced phosphorylation of proteins regulating or regulated by the mTORC1 pathway. Cells were incubated for 17 h with 1000 ng/ml tunicamycin or 1000 nM thapsigargin before stimulation with 10 nM or 50 nM insulin for 15 min (A) or with 2.5 mM or 5 mM leucine for 30 min (B). (C) Phosphorylation of S6K1 (Thr389) and PRAS40 (Thr246) after PKB inhibition and/or leucine stimulation. Cells were serum-starved for 16 h, pre-incubated with 100 nM, 500 nM or 1 µM PKB inhibitor (Akti) for 1 h and stimulated with 2.5 mM or 5 mM leucine in combination to Akti or not for 30 min. TN, tunicamycin; TG, thapsigargin; PKB, protein kinase B; PRAS40, proline-rich Akt substrate 40 kDa; GSK3, glycogen synthase kinase-3; PDK1, phosphoinositide-dependent kinase 1; mTOR, mammalian target of rapamycin; S6K1, ribosomal protein S6 kinase 1; 4E-BP1, eukaryotic initiation factor 4E-binding protein 1; eEF2, eukaryotic elongation factor 2; Ins, insulin; Leu, leucine.

After determining that leucine uptake was not limiting, we next established the point at which ER stress interrupted the activation of mTORC1, by assessing changes in the phosphorylation of S6K1 on Thr389. As with the Ser473 site, basal phosphorylation of PKB at Thr308 was decreased following treatment with tunicamycin (1000 ng/ml) and thapsigargin (1000 nM). Tunicamycin decreased Thr308 phosphorylation in response to both low and high levels of insulin. The inhibitory action of thapsigargin was less potent, decreasing Thr308 phosphorylation at low but not high levels of insulin. Even though tunicamcyin and thapsigargin decreased PKB phosphorylation, the phosphorylation of PRAS40 on Thr246 was still enhanced by insulin, indicating that following insulin stimulation tunicamycin and thapsigargin did not repress mTORC1 via PRAS40. As with Thr389, the phosphorylation of S6K1 on Thr421/Ser424 mirrored that of PKB. When PKB phosphorylation was decreased, S6K1 phosphorylation followed suit (Thr389; Figure 3 and Thr421/Ser424; Figure 5). Neither PDK1, p38, ERK1/2, nor JNK phosphorylation was decreased by either tunicamycin or thapsigargin (data not shown).

Since activation of mTORC1 by amino acids generally occurs independent of PKB, it was not surprising that PKB phosphorylation was not changed by the addition of leucine (Figure 5B). As described above, tunicamycin and thapsigargin decreased basal PKB phosphorylation and this was also true after leucine treatment. The phosphorylation of PRAS40 on Thr246 followed the same pattern, completely absent in the presence of either tunicamycin or thapsigargin. As expected, in untreated cells leucine increased the phosphorylation of S6K1 on Thr421/Ser424 and 4EBP1 on Thr37/46. Tunicamycin and thapsigargin completely prevented both of these leucine-induced changes. As mentioned in the introduction, activation of 4E-BP1 is a two-step event that first requires the phosphorylation of Thr37/46. When 4E-BP1 is less active after treatment with tunicamycin or thapsigargin, the phosphorylation state of Thr37 and Thr46 remains high. Under serum-starvation conditions, Thr37 and Thr46 phosphorylation can remain high [16]. Following a second stimulus, such as insulin, 4E-BP1 becomes phosphorylated on Ser65 and Ser70, with a small decrease in phosphorylation of Thr37 and Thr46 being observed [39]. Based on the literature [39], a shift towards less fast migrating bands with insulin can be interpreted as a higher phosphorylation state of 4E-BP1 and a shift towards faster migrating bands with tunicamycin and thapsigargin as a lower phosphorylation state.

We surmised that the low phosphorylation of PKB and PRAS40 resulted in the allosteric inhibition of mTORC1 in response to leucine. To test this hypothesis, PKB activity was blocked chemically [40] and the resulting effect on PRAS40 and leucine stimulation of S6K1 was determined (Figure 5C). Consistent with our hypothesis, in the presence of the PKB inhibitor neither PRAS40 nor S6K1 were phosphorylated, indicating that PKB is permissive for the activation of mTORC1 by amino acids (Figure 5B and [7]).

In summary, whereas ER stress only partially prevents insulin stimulation of PKB and mTORC1, the lack of activation of PKB by amino acids results in a complete block of leucine-stimulated activation of targets downstream of mTORC1 likely through hypophosphorylation of the mTORC1 kinase inhibitor PRAS40.

## Discussion

The main finding of the present study is that ER stress induces anabolic resistance in muscle cells through PKB/PRAS40-induced blockade of mTORC1. Anabolic resistance is defined as the inability to increase protein synthesis in response to an increase in amino acids following a meal [9] or other anabolic stimuli [10], [41]. The molecular mechanisms behind anabolic resistance are not well understood although decreased expression and phosphorylation of amino acid sensing/signaling proteins, such as mTORC1 and S6K1, seem to be involved [10]. The present study has identified the inactivation of PKB as a likely mechanism underlying ER stress-induced anabolic resistance. High levels of insulin, which continue to activate PKB under ER stress, albeit to a lesser degree, can partially overcome anabolic resistance. However, since amino acids do not normally activate PKB, the competitive inhibitor of mTORC1, PRAS40, remains hypophosphorylated and prevents the phosphorylation of mTORC1 targets such as S6K1 and 4EBP. This phenotype is replicated by the PKB inhibitor Akti suggesting that basal PKB activity is required for amino acid induced activation of mTORC1. We also show that the effect of ER stress on mTORC1 is dose-dependent since the rise in BiP expression occurred proportionally with a decrease in PKB and S6K1 phosphorylation. This relationship suggests that part of the ER stress response is to block PKB and mTORC1 in an effort to decrease protein synthesis.

It should be mentioned that when cells were stimulated with insulin, this was made in presence of basal levels of leucine (0.8 mM) usually found in cell culture medium, whereas when cells were stimulated with extra leucine (2.5 or 5 mM), there was no insulin or serum. As insulin is known to play a permissive role in the action of amino acids on the mTORC1 pathway, it is possible that the effect of additional leucine was relatively less optimal compared to insulin. This could have contributed to the fact that ER stress was more potent in reducing leucine- than insulin-induced stimulation of mTORC1.

Having previously established a negative relation between ER stress and the mTORC1 pathway [28], the current work focussed on determining the mechanism underlying this effect. We found that two inhibitors of PKB/mTORC1, TRB3 [36] and REDD1 [25], were transcriptionally increased by ER stress. Even though both of these inhibitors could be increased by ER stress, the effect on REDD1 was minor and only seen with thapsigargin, indicating that REDD1 was not a key player in ER stress-induced anabolic resistance. TRB3 on the other hand was increased over 30-fold by both ER stress agents. TRB3 directly binds to and inhibits the kinase activity of PKB [36]. In our basal conditions, PKB activity, as determined by phosphorylation of PRAS40 on Thr246, was decreased consistent with the published effects of elevated TRB3. Recently, Chen et al showed that both TN and TG could also increase the phosphorylation of rictor at Ser1235 [42]. The phosphorylation of rictor on this residue, like TRB3, decreases mTORC2 activity towards PKB at Ser473, and is completely consistent with the decrease in PKB and mTORC1 activity we describe here. However, insulin stimulation could partially or even totally reverse the physiologic blockade of PKB, indicating that the effect of TRB3 is reversible. The situation is different with leucine, as amino acids do not activate PKB in myotubes. We speculated that if PKB activity was decreased and PKB was permissive in mTORC1 activation by amino acids, that leucine would be unable to activate S6K1 and 4EBP downstream of mTORC1. Consistent with this hypothesis, ER stress prevented S6K1 and 4EBP phosphorylation in response to leucine. The fact that this effect was due to inhibition of PKB was confirmed using a specific inhibitor: Akti [40]. Both ER stress and Akti decreased PRAS40 phosphorylation as well as that of S6K1 and 4EBP. PKB phosphorylates PRAS40 on Thr246 resulting in the unmasking of a TOS motif that allows the phosphorylation of PRAS40 on Ser183 and Ser221 by mTORC1 [22]. Together, these phosphorylation events lead to disassociation from raptor in favor of binding to 14-3-3. The dissociation of PRAS40 allows mTORC1 to bind to its downstream targets such as S6K1 and 4EBP [22]. These data show that a permissive level of PKB activity, likely through phosphorylation of PRAS40, is necessary for leucine to increase the phosphorylation of S6K1 and 4EBP by mTORC1. This is in agreement with recent data from the heart where phosphorylation of PRAS40 is required for leucine to increase S6K1 activity [43]. However, using a knockin mutation where PDK1 phosphorylation of PKB is maintained but S6K activation is prevented, they showed that the phosphorylation of PRAS40 was prevented along with leucine signaling to S6K1 [43]. These data suggest that there may be another PRAS40 kinase responsible for anabolic resistance in response to ER stress.

Another possible mechanism of anabolic resistance is a decrease in amino acid transporter levels and/or amino acid uptake. Several transporters are known to participate directly or indirectly in leucine transport in muscle cells. The preferred transporter for leucine is the system L-type [44] which is sodium-independent. The L-type transporter is a heterodimeric complex made up of a light subunit, LAT1 or LAT2, that functions as an amino acid permease and a glycosylated heavy subunit, 4F2hc also known as CD98 [45]. Another class of amino acid transporters that plays an indirect role in leucine transport is the sodium-dependent system A-type, which includes SNAT2 and ASC1. LAT1-4F2hc and SNAT2 are involved in muscle growth and their expression has been correlated with mTORC1 activation [4], [46], whereas inhibitors of SNAT2 and 4F2hc reduce mTORC1 activity and protein synthesis [47], [48]. SNAT2 and LAT1-4F2hc work together to increase leucine uptake in a process called tertiary active transport. Tertiary active transport uses the sodium gradient established by the Na/K ATPase to drive sodium-dependent uptake of glutamine through SNAT2. Once in the cell, glutamine exchange for leucine through LAT1-4F2hc increases the influx of leucine [49]. Consistent with the hypothesis that ER stress decreased leucine uptake, LAT1 mRNA was decreased by thapsigargin and tunicamycin. However, this did not lead to a decrease in leucine uptake. The maintenance of leucine transport in the face of decreased LAT1 mRNA may be due to the compensatory increase in 4F2hc and SNAT2. An increase in 4F2hc would mobilize any LAT1 protein to the membrane and an increase in SNAT2 would provide a larger glutamine gradient to enhance the exchange properties of any LAT1-4F2hc transporters that were at the membrane. However, whether prolonged ER stress would result in decreases in leucine uptake still needs to be determined. As mTORC1 can regulate LAT1 expression [46], a decrease in mTORC1 activity probably participated in the decreased LAT1 mRNA expression. In summary, the anabolic resistance following acute ER stress is not due to a decrease in amino acid uptake as the latter tended to increase. This is consistent with *in vivo* studies that have shown that anabolic resistance is not due to reduced amino acid availability [41].

In C2C12 muscle cells, ER stress seems to impair mTORC1 rather than *vice versa* [26], [27]. We have previously shown that hyperactivation of mTORC1 by insulin for 6 h or 24 h did not trigger the unfolded protein response whereas tunicamycin activated the unfolded protein response before S6K1 phosphorylation decreased, suggesting that in C2C12 muscle cells the induction of ER stress precedes the impairment in mTORC1 activity [28].

The present study suggests that ER stress is a potential mediator of the anabolic resistance observed in the muscle of aged [10], immobilized [11], and obese [12] animals. *In vitro*, ER stress completely blocks any effect of amino acids on S6K1 and 4EBP phosphorylation, whereas the effect on insulin was less dramatic. This is in accordance with the fact that anabolic resistance *in vivo* is mainly due to decreased sensitivity to amino acids and impaired mTORC1 signaling, rather than an impaired response to insulin [9]. In conclusion, the present study shows that ER stress induces anabolic resistance in muscle cells through a PKB-dependent, PRAS40-induced, blockade of mTORC1.
